# Multiscale Thermal Technologies: Exploring Hot and Cold Potentials in Biomedical Applications

**DOI:** 10.3390/bioengineering11101028

**Published:** 2024-10-15

**Authors:** Bangrui Yu, Haishui Huang

**Affiliations:** 1 Bioinspired Engineering and Biomechanics Center (BEBC), School of Life Science and Technology, Xi’an Jiaotong University, Xi’an 710049, China; 2 The Key Laboratory of Biomedical Information Engineering of Ministry of Education, Xi’an Jiaotong University, Xi’an 710049, China

Harnessing thermal technology has opened up new possibilities in biomedicine in areas such as cancer treatment, biopreservation, and assisted reproduction. Thermal technology applied at the macro, micro, and nano scales is driving innovation in these various fields, but some challenges must be overcome to fully realize its clinical and research potential. This Special Issue compiles groundbreaking research on cryopreservation and thermal therapy, including six original articles and five review papers that improve our ability to control thermal environments and address critical issues such as cryogenic damage, precise heat transfer, and biocompatibility. This Special Issue sets the stage for the next wave of breakthroughs in thermal engineering by integrating insights from biology, materials science, and thermodynamics.

Cryopreservation is a cornerstone of modern biomedicine, enabling the preservation and future use of living cells, tissues, organs, and biomolecules such as proteins and mRNA [1]. However, cryopreservation technology still faces major hurdles, particularly in controlling ice formation during cooling and recrystallization during warming, both of which can cause mechanical and osmotic damage [2]. High concentrations of cryoprotectants (CPAs) can reduce ice formation via inducing vitrification, although these agents are often cytotoxic [3,4]. In addition, cryodamage, including apoptosis, necrosis, and ischemia-reperfusion injury, remains a critical challenge in ensuring the viability of preserved biological samples [5].

Several of the innovative approaches presented in this Special Issue offer solutions to these challenges. Dou et al. present liquid helium as a novel cryogenic medium for stem cell vitrification that offers substantially faster cooling rates than conventional liquid nitrogen [6]. Liquid helium reduces ice formation and increases survival after thawing, providing a more efficient and scalable solution for cell storage in regenerative medicine. Amini and Benson comprehensively overviewed vitrification techniques, emphasizing the urgent need for less-toxic CPAs and scalable protocols, especially for larger tissues [7]. Johnson et al. conducted a detailed analysis of crystallization kinetics in solution-containing solutions, proposing optimized freezing models that minimize the damage caused by ice [8].

Natural alternatives to synthetic CPAs are also being studied. Cheepa et al. used honey as a non-toxic, naturally occurring cryoprotectant, demonstrating its efficacy in reducing oxidative stress and inhibiting ice crystal formation in sperm, oocytes, and embryos [9]. Similarly, Azevedo et al. used medical-grade honey (MGH) for ovarian tissue transplantation. MGH promotes angiogenesis and mitigates ischemic injury, positioning honey as a viable, cost-effective option for fertility preservation, particularly in people with cancer [10].

Despite these advances, cryoinjury remains a major challenge. Fu et al. investigated the molecular pathways of cryoinjury and propose strategies to mitigate its effects, such as improving mitochondrial regulation and reducing reactive oxygen species (ROS) production [11]. These findings provide a solid theoretical foundation for future research aimed at further improving the survival and functionality of cryopreserved cells and tissues. Ultimately, the further optimization of CPA formulations, freezing protocols, and molecular damage control is essential for fully exploiting the potential of cryopreservation in clinical and research applications.

In addition to cryopreservation, thermal technology has shown promise in heat-based applications, particularly in cancer therapy. Techniques such as photothermal therapy (PTT) and thermotherapy enable the targeted destruction of diseased tissues while minimizing damage to healthy cells [12]. However, efficient heat transfer, the precise alignment of nanoparticles, and long-term biocompatibility remain critical barriers to clinical implementation [13,14,15].

In this Special Issue, Dai et al. provide an overview of synergistic thermal therapies for cancer, focusing on the combination of thermal therapy with immunotherapy, chemotherapy, and other treatment modalities to increase therapeutic efficacy [16]. Their review highlights the current challenges in optimizing dosing and targeting and provides a roadmap for future research on combination therapies. Taylor et al. present the developments in gold-nanorod-assisted photothermal therapy (AuNR-PTT) [17]. With AuNR-PTT, the tunable optical properties and robust photothermal conversion efficiency of gold nanorods are used to provide enhanced light absorption and scattering through localized surface plasmon resonance (LSPR). The researchers also explored the potential for combining AuNR-PTT with chemotherapy, where gold nanorods serve not only as photothermal agents but also as carriers for chemotherapeutic drugs, creating a powerful dual-action treatment platform.

Thermal technology in biomedical applications influences far more than the known applications in cancer treatment. In physiological monitoring and intervention, thermal models play a crucial role in improving diagnostics and therapeutic outcomes. For example, Ding et al. developed an integrated thermofluid model with lumped parameters to assess hemodynamic changes during human fatigue states [18]. Their findings demonstrate how cardiovascular changes, particularly peripheral resistance and heart rate, directly influence skin temperature, providing a noninvasive method for monitoring fatigue. This model has considerable potential for fatigue detection and monitoring applications in areas such as road safety and occupational health, where the accurate and early detection of fatigue is critical. Their results demonstrate the importance of understanding the heat transfer processes in different tissues to optimize the effectiveness of real-time monitoring systems.

Thermal technology also shows considerable potential for the treatment of cardiovascular diseases. Wang et al. developed a novel approach for the ablation of atherosclerotic plaques by combining volumetric radiofrequency heating with convective cooling [19]. This method enables precise plaque removal while sparing the surrounding healthy tissue and thus offers a solution for plaques of varying thickness. Campelo et al. advanced their work on real-time temperature monitoring during irreversible electroporation (IRE), a minimally invasive technique used for the treatment of tumors and vascular lesions [20]. Using state-space modeling, they developed a method for predicting temperature changes during IRE with high accuracy, allowing for the more precise control of thermal effects and minimizing damage to nontarget tissue. These advances pave the way for the safer and more reliable application of IRE in clinical settings.

Thermal technology has rapidly emerged as a transformative force in biomedicine, offering breakthrough solutions across a spectrum of applications from cryopreservation to advanced thermotherapy. The research featured in this Special Issue underscores the considerable potential of integrating biology, materials science, and thermodynamics in addressing challenges in both cold and hot biomedical contexts. Remarkable progress has been achieved in areas such as tissue preservation, targeted cancer therapy, and cardiovascular interventions through these interdisciplinary approaches. However, despite these promising advances, numerous critical obstacles remain prior to clinical translation, including optimizing nanoparticle targeting to enhance specificity, minimizing cellular and molecular damage during cryogenic storage, and ensuring the long-term biocompatibility and safety of heat treatments. Additionally, the scalability and standardization of these methods across different biological systems must be achieved for their widespread adoption in clinical practice. Overcoming these barriers will unlock the full therapeutic potential of thermal engineering, enabling its successful integration into clinical and translational medicine, ultimately leading to improved patient outcomes across a wide range of conditions.
